# Supplementary material for: Mapping the evolution of fertility support policies in China: A content and instrumental analysis
Source: PLoS One. 2025 Oct 9;20(10):e0332137. doi: 10.1371/journal.pone.0332137 (PMC12510515; doi:10.1371/journal.pone.0332137)
Supplement: S1 Appendix — (ZIP) [file pone.0332137.s001.zip › S1 Appendix. 226 original policy documents/24-国家卫生计生委关于印发2016年卫生计生工作要点的通知(FBM-CLI-4-263401).doc]

国家卫生计生委关于印发2016年卫生计生工作要点的通知

**发布部门：** [国家卫生和计划生育委员会(已撤销)](https://www.pkulaw.com/law/chl?Aggs.IssueDepartment=60319) [机构沿革](javascript:void(0))

**发文字号：**国卫办发〔2016〕6号

**发布日期：**2016.01.19

**实施日期：**2016.01.19

**时效性：** [现行有效](https://www.pkulaw.com/law/chl?Aggs.TimelinessDic=01)

**效力级别：** [部门工作文件](https://www.pkulaw.com/law/chl?Aggs.EffectivenessDic=XE0303)

**法规类别：** [卫生综合规定](https://www.pkulaw.com/law/chl?Aggs.Category=09601)

国家卫生计生委关于印发2016年卫生计生工作要点的通知
（国卫办发〔2016〕6号）

各省、自治区、直辖市卫生计生委，计划单列市及新疆生产建设兵团卫生计生委（卫生局、人口计生委），委机关各司局，委直属和联系单位：
　　《2016年卫生计生工作要点》已由国家卫生计生委第76次委主任会议讨论通过，现印发给你们（可从国家卫生计生委网站下载）。请结合实际，研究制定本地区、本司局和本单位的工作要点，切实加强组织领导，建立任务清单和责任清单，严肃考核问责，确保完成各项任务。

国家卫生计生委
2016年1月19日

　　2016年卫生计生工作要点

　　2016年是“十三五”开局之年，也是全面贯彻落实十八届五中全会精神，加快推进健康中国建设，促进人口均衡发展的重要一年。卫生计生工作的总体要求是：高举中国特色社会主义伟大旗帜，以邓小平理论、“三个代表”重要思想、科学发展观为指导，全面贯彻落实党的十八大、十八届二中、三中、四中、五中全会精神和习近平总书记系列重要讲话精神，加快医药卫生体制改革，稳妥有序实施全面两孩政策，进一步健全全民医疗保障体系，进一步完善医疗卫生服务体系，进一步加强公共卫生和重大疾病预防控制，大力促进中医药事业发展，大力推动医药科技创新，大力发展健康服务业，实现卫生计生事业科学发展。重点做好以下工作：
　　**一、**加快推进医药卫生体制改革
　　编制医改“十三五”规划，坚持“保基本、强基层、建机制”的基本原则，完善医药卫生管理、运行、投入、价格政策，强化医疗、医药、医保联动，更加注重改革的系统性、整体性、协同性，推动重心下移和资源下沉，推动重点领域和关键环节的改革取得突破性进展和明显成效。
　　（一）全面深化公立医院综合改革。巩固扩大完善三级试点，巩固县级公立医院综合改革，加强分类指导，开展改革效果评估，树立若干个综合改革示范县。城市公立医院综合改革试点城市增加到200个，试点区域内所有公立医院纳入改革范围整体推进。增加综合医改试点省份，发挥连片带动效应。参与改革的公立医院取消药品加成（中药饮片除外），健全调整医疗技术服务价格和增加政府补助、医院加强核算、节约运行成本等多方共担的补偿机制。按照“总量控制、结构调整、有升有降、逐步到位”的原则和“腾空间、调结构、保衔接”的步骤，加强与药品采购、医保支付、薪酬制度、分级诊疗等政策的衔接。落实政府投入责任，研究制订公立医院债务化解的政策措施。制订现代医院管理制度框架文件，以加快建设公立医院运行新机制为核心，理顺医药价格体系，建立符合行业特点的人事薪酬制度，落实公立医院人事管理、内部分配、运营管理等自主权，建立健全公立医院综合性绩效考核制度，强化院长年度和任期目标管理。深化编制人事制度改革，会同中央编办开展公立医院编制管理创新研究，逐步实行编制备案制。制订委属委管医院绩效考核的指导性文件，推进委属委管医院参与属地公立医院综合改革。
　　（二）健全全民基本医保体系。继续提高城乡居民基本医保人均财政补助标准，建立健全稳定可持续筹资和待遇水平报销比例调整机制，政策范围内报销比例稳定在75%左右。城乡居民大病保险人均筹资进一步提高，支付比例提高至50%以上。鼓励各地结合实际、量力而行，适当扩大合规医疗费用范围，探索对贫困人口的倾斜政策。完善疾病应急救助制度，做好急危重伤病患者的紧急救治，对符合条件的身份不明或者无力支付医疗费用患者提供救助资金保障。整合城乡居民基本医疗保险制度，逐步统一覆盖范围、筹资政策、保障待遇、医保目录、定点管理和基金管理。鼓励商业保险机构参与经办服务。制订政策措施，加大医保支付方式改革力度，实行按病种、按人头、按服务单元、按疾病诊断相关组等复合型支付方式，控制医药费用不合理增长。持续拓展新农合跨省就医核查和异地即时结报试点。推动建立基本医保、城乡居民大病保险、疾病应急救助、医疗救助等制度的信息互通和操作衔接机制。
　　（三）加快推进分级诊疗制度建设。优化医疗卫生机构布局，合理配置医疗资源，健全上下联动、衔接互补的医疗服务体系。在70%左右的地市开展分级诊疗试点，综合医改试点省和公立医院改革试点城市要全面推开。制订规范城市及县域内医疗联合体建设的政策措施，明确分工，推动形成利益和责任共同体，贯通上下联动。提升县医院综合能力，完善基层医疗服务模式，扩大二级以上医院医师与基层医疗卫生机构的医务人员组成团队签约服务试点。制订指导急慢分治工作的指导性文件，继续扩大慢性病分级诊疗试点工作。
　　（四）巩固完善基层运行新机制。健全多渠道补偿机制，落实基层医疗卫生机构基本建设、设备购置经费。提高统筹力度，合理使用公共卫生服务资金、基本药物制度补助资金，完善医保支持政策和价格政策，探索完善基层财务收支管理制度。推进基层人事分配制度改革，强化绩效考核，调动基层医务人员积极性。落实乡村医生补偿、养老相关政策，稳定乡村医生队伍。继续开展基层卫生综合改革重点联系点工作，深入开展建设“群众满意的乡镇卫生院”活动和社区卫生服务提升工程，组织开展全国基层卫生技能竞赛。促进基本公共卫生服务均等化。继续提高基本公共卫生服务经费财政补助标准，新增部分全部用于全科医生（乡村医生）签约服务。健全基层医疗卫生机构与专业公共卫生机构分工协作机制，继续扩大服务受益面，提高服务成效。开展流动人口健康促进行动，提高流动人口基本公共卫生计生服务均等化水平。
　　（五）健全药品供应保障机制。全面实施公立医院药品集中采购和高值医用耗材网上阳光采购，鼓励和引导跨区域联合采购，切实降低虚高药价。增加国家药品价格谈判药品品种，降低专利药品和独家生产药品价格。扩大国家定点生产药品范围。配合推动小品种药品生产基地建设。建立生产、配送企业约谈制度，完善低价、短缺药品配送管理，重点提高乡村、边远地区药品配送能力。推动保障儿童和老年人基本用药工作。开展基本药物和儿童用药临床综合评价体系建设。进一步完善国家药品供应保障综合管理信息平台和省级药品集中采购平台规范化建设，建立预警机制。
　　（六）完善人才培养评价机制。推进医教协同，构建以“5＋3＋X”为主体的临床医学（含中医学）人才和以全科医生为重点的基层卫生人才培养体系，全面实施住院医师规范化培训制度，继续开展第三方评估。开展专科医师规范化培训制度试点和公共卫生医师规范化培训。启动“3＋2”助理全科医生培训试点。进一步改革完善基层卫生专业技术人员职称评审工作，健全基层卫生人才考评机制。加强精神医学、产科、儿科、康复、病理、药学、老年医学、老年护理等紧缺专业人才培养，深入推进继续医学教育。实施卫生计生经济管理人才队伍建设工程、中医药传承与创新人才工程。
　　（七）促进健康服务业发展。进一步优化社会办医的发展环境，落实同等待遇，优先支持社会力量举办非营利性医疗机构，加快形成多元化办医格局。简化医师多点执业程序，促进优质医疗资源平稳有序流动。实施健康老龄化工程，启动医养结合项目试点，大力发展健康养老等生活性服务业，推动发展护理、康复、临终关怀等延伸服务，完善政策措施，推动国产医用设备发展应用，积极发展医疗旅游。
　　（八）加快卫生计生信息化建设。统筹推进国家、省、地市、县级人口健康信息平台建设，建立公共卫生、计划生育、医疗服务、医疗保障、药品管理、综合管理业务应用信息系统，尽快形成全国人口信息库，促进互联互通、业务协同。启动实施全民健康保障信息化工程一期项目，推进电子健康档案和电子病历在各级各类医疗机构之间授权使用。积极发展远程医疗、疾病管理等网络业务应用，整合健康管理及医疗信息资源，推动预约诊疗、线上支付、在线随访以及检查检验结果在线查询等服务。开展健康医疗大数据应用发展试点示范工作，积极实施“互联网＋健康医疗”服务，通过信息化手段，放大群众的获得感。

　　**二、**坚持计划生育基本国策，促进人口均衡发展
　　（九）稳妥实施全面两孩政策。贯彻落实《[关于实施全面两孩政策改革完善计划生育服务管理的决定](https://www.pkulaw.com/chl/8564756bae805261bdfb.html)》，推动修订《[社会抚养费征收管理办法](https://www.pkulaw.com/chl/f03446d289d2b59ebdfb.html)》《[流动人口计划生育工作条例](https://www.pkulaw.com/chl/f6b2c35d55b7a40ebdfb.html)》等法律法规，指导各地做好人口与计划生育条例的修订工作，完善配套衔接政策，维护群众合法权益。深入开展政策解读，加强宣传倡导，强化风险防控，做好信访维稳。加快全国出生医学证明管理信息系统建设，实现出生人口信息互联共享。开展政策实施情况效果评估，加强人口与计划生育形势分析。
　　（十）提升妇幼健康服务保障能力。加强妇幼健康服务体系建设，优化整合妇幼健康和计划生育服务资源，扩充产科儿科床位，加大产科、儿科和助产士专业人才培养力度。重点强化危重孕产妇救治与新生儿救治中心能力和网络建设，确保母婴安全。开展妇幼健康服务机构评审，持续推进妇幼健康优质服务示范工程，创新爱婴医院品牌，加强助产技术及人类辅助生殖技术监管，推广中医妇科、儿科适宜技术。落实计划生育免费基本技术服务，加强再生育咨询服务和技术指导，完善避孕药具管理。继续实施妇幼健康重大专项。做好优生优育全程服务，整合孕产期保健、儿童保健、预防接种、计划生育等服务内容，扩大母子保健手册使用试点。促进儿童早期发展，加强出生缺陷综合防治，国家免费孕前优生健康检查项目覆盖所有城乡居民，提高出生人口素质。
　　（十一）深化计划生育服务管理改革。稳定和加强基层计划生育网络和队伍，提高服务管理水平，实行生育登记制度，两孩生育不再审批。开展流动人口婚育信息网络异地查询，取消纸质婚育证明，推行网上办事，优化办事流程，实现国家和省级计划生育业务信息互联互通。完善流动人口统计监测，促进社会融合。坚持和完善计划生育目标管理责任制，落实一票否决制。深入开展诚信计生活动和新一轮全国计划生育优质服务先进单位创建活动并进行评选表彰。
　　（十二）促进计划生育家庭发展。完善计划生育家庭奖励和扶助政策，做好全面两孩政策实施后的政策衔接。深入实施农村部分计划生育家庭奖励扶助制度、计划生育家庭特别扶助制度和西部地区“少生快富”工程，加大对计划生育特殊家庭扶助关怀力度。全面开展创建幸福家庭活动，深入实施新家庭计划、家庭科学育儿、青少年健康发展、养老照护试点项目，健全家庭健康服务体系，做好家庭发展追踪调查。加大出生人口性别比综合治理力度，深入开展“关爱女孩行动”和“圆梦女孩志愿行动”，推动整治“两非”工作取得实效。

　　**三、**加强公共卫生和重大疾病防控工作
　　（十三）提高卫生应急核心能力。制订卫生应急工作规范化建设指导意见，指导支持基层卫生应急队伍专业化建设，完善联防联控指挥协调机制，推进突发公共卫生事件综合检测、风险评估和及时预警，启动陆海空立体化紧急医学救援网络建设。做好突发公共卫生事件和重特大自然灾害、事故灾难、社会安全事件的卫生应急工作。
　　（十四）有效防控重大疾病。完善重大疾病防控策略。探索与世界卫生组织及相关国家建立重大疫情防控沟通协作机制，有效防范境外传染病输入。落实重点传染病防控年部署、季调度、月评估，有效控制暴发流行。完善社会组织参与艾滋病防治基金项目监督管理和评价机制，开展结核病综合防治模式、精神卫生综合管理试点，增加艾滋病、结核病、严重精神障碍治疗等特殊药物免费供给。强化预防接种，积极推进全国使用二价脊髓灰质炎减毒活疫苗替代工作，开展预防接种异常反应补偿保险试点。完成上海等省（市）消除血吸虫病复核工作。加强国家和省级慢性病综合防控示范区建设，提高对高血压、糖尿病患者的管理干预水平，对高危人群开展癌症等疾病早诊早治工作。加强地方病、职业病防治工作，开展包虫病综合防治试点，强化尘肺病防治工作。发布新版中国居民膳食指南，在全国1/3的县开展人群碘营养监测，引导做好科学补碘。制订进一步加强疾控体系建设的指导意见，推动落实疾控中心机构编制指导意见、疾控中心岗位设置指导意见和传染病防治人员安全防护意见。探索开展疾控机构规范化建设综合评价。
　　深入开展爱国卫生运动，完善卫生城镇创建长效管理机制，全面启动健康城市建设，加大城乡环境卫生整洁行动督导检查力度，继续实施农村改厕工作。扩展城乡居民饮用水、空气污染（雾霾）对人群健康影响和病媒生物监测覆盖面。
　　（十五）加强食品安全标准建设和风险监测评估。贯彻落实《[食品安全法](https://www.pkulaw.com/chl/be352ba1d170a5a8bdfb.html)》，健全工作体系，修订完善[食品安全法实施条例](https://www.pkulaw.com/chl/0c8f8fee347bbebebdfb.html)及相关配套文件。完善食品安全国家标准体系，制修订一批重点亟需的食品安全国家标准，组织开展标准的宣传、贯彻与跟踪评价。全面落实2016年国家食品安全风险监测计划，加强食源性疾病监测报告以及溯源能力建设。改革和加强食品“三新产品”管理，开展食品安全风险评估方法研究和基础数据收集，组织做好年度计划评估和应急评估工作。推进建设县乡村一体化基层食品安全网络，加强信息化建设，促进食品安全信息惠民项目。

　　**四、**进一步提升医疗质量和服务水平
　　（十六）切实改善医疗服务。印发《医疗质量管理办法》，开展提升医疗质量专项活动。建立医疗质量管理与控制长效机制，强化医疗技术临床应用的事中事后监管。推进医疗机构、执业医师、护士注册信息联网公开。启动乡村全科执业助理医师资格考试试点。推动药师立法和药师管理，加强临床药事管理。实施遏制细菌耐药国家战略。提升患者安全管理水平，加强药品和医疗器械临床使用管理。遴选适宜病种临床路径在三级医院和县医院推广实施，加大临床路径管理培训力度。开展肿瘤等重大疾病规范化诊疗管理，修订《医疗技术临床应用管理办法》。巩固血站血液核酸检测全覆盖的成果，健全血液质量控制和改进体系，建立以单病种为主的临床用血考核评价制度，保证血液质量和安全。提升保健服务能力，做好重大会议、活动的医疗保障。实施进一步改善医疗服务行动计划，逐步推开日间手术，落实便民惠民利民措施，提高群众获得感。加强护士队伍建设，持续推进优质护理服务。保持对涉医违法犯罪行为严厉打击的高压态势，完善医疗纠纷调解和处置长效机制，构建和谐医患关系。
　　（十七）实施健康扶贫工程。建立农村贫困人口健康卡，开展签约服务，实行“两提高、两降低”倾斜政策，提高新农合门诊报销水平，提高政策范围内报销比例，增长幅度不低于5个百分点；降低病残儿童、重度残疾人以及大病保险报销起付线，降低贫困人口大病费用个人实际支出。落实对口帮扶机制，实施全国三级医院对贫困县县级医院稳定持续的“组团式”帮扶，提升贫困县县医院水平，基本满足县域内居民基本医疗服务需要。加大贫困地区重点疾病防控力度，适宜重大公共卫生服务项目覆盖所有贫困地区。采取有针对性的措施，加强革命老区、少数民族地区健康扶贫工作，进一步支持西藏及四省藏区、新疆等地区卫生计生事业发展。

　　**五、**促进中医药事业发展
　　（十八）深化中医药改革。出台实施《中医药发展战略规划纲要（2015-2030年）》。完善中医药事业发展政策和机制。同步推进公立中医医院改革，完善差别化的中医医院改革政策，提升县级中医医院综合服务能力，推动分级诊疗制度建设。深化中医药教育综合改革和科技体制改革。加强国家中医药综合改革试验区建设。
　　（十九）提升中医药事业发展能力。促进中医药健康服务业发展升级，大力发展中医“治未病”服务，推动中医药旅游示范区建设，培育新兴业态。做好名老中医药专家学术经验与古籍文献传承研究工作，加紧编撰出版《中华医藏》。加强中药资源保护，推进全国第四次中药资源普查。实施基层医疗卫生机构“中医馆”建设，持续提升基层中医药服务能力。加强国家中医临床研究基地及省级中医药科研院所建设。提升地市级以上中医医院科研能力。助推中医药参与“一带一路”建设，推动建设中医药海外中心，开展中医药海外惠侨、义诊和文化宣传工作。推动中西医结合和民族医药事业发展。

　　**六、**加强卫生计生规划与法治建设
　　（二十）编制实施健康中国建设相关规划。从大健康、大卫生的高度出发，坚持以问题为导向，以五大发展理念为引领，研究编制推进健康中国建设的纲领性文件，切实加强对国民健康重大和长远问题的有效干预。科学设定规划的目标指标，确定一批重大政策、重大工程和重大项目，以较少的社会成本实现较高的健康产出。同时，编制实施“十三五”卫生与健康、医改、中医药事业发展、京津冀医疗卫生协同发展、卫生计生服务体系等各专项建设和重大疾病防治等专项规划。
　　（二十一）推进依法行政。加强综合性卫生计生政策研究，积极配合全国人大完成《基本医疗卫生法》草案的起草、征求意见，年底形成草案报全国人大常委会，配合做好《中医药法》《医疗纠纷预防与处理条例》等法律法规制修订工作，加快制修订卫生计生事业发展亟需的部门规章，强化规范性文件合法性审查，结合行政审批制度改革清理修订相关部门规章和规范性文件。继续清理行政审批事项，规范行政审批程序，坚持放管结合，强化事中事后监管。完善公共政策专家咨询委员会工作机制。加强人口健康政策法规评估系统建设。落实国务院《深化标准工作改革方案》，梳理完善各专业卫生标准体系。
　　（二十二）推进卫生计生监督执法体系建设。落实6部门[关于进一步加强卫生计生综合监督行政执法工作的意见](https://www.pkulaw.com/chl/e7f0977dcbe61e97bdfb.html)，开展培训和监督工作。规范行政执法流程，严格执法稽查，开展案例评查。开展《[人口与计划生育法](https://www.pkulaw.com/chl/18c6c388fc14eaa9bdfb.html)》《[职业病防治法](https://www.pkulaw.com/chl/d77630c247f766f9bdfb.html)》等法律法规落实情况监督检查，开展“双随机”抽查机制试点。组织开展国家监督抽检，做好监督信息公开，加强社会监督。围绕关系群众利益的重点难点问题，开展干细胞临床应用和计划生育行政执法工作等专项监督检查。推进医疗卫生机构传染病防治分类监督综合评价试点工作。督查督办卫生计生重大违法案件。

　　**七、**为事业改革发展提供强大保障
　　（二十三）加强思想政治、行风建设与反腐倡廉工作。深入学习党的十八大、十八届二中、三中、四中、五中全会精神和习近平总书记系列重要讲话精神，引导全系统干部职工将思想和行动统一到党中央、国务院的决策部署上来。加强理想信念和宗旨教育，开展纪律教育。以全国文明单位和青年文明号创建为抓手，推进行业精神文明建设和医院文化建设。发挥群团组织优势，最大限度为改革发展凝聚共识和力量。落实全面从严治党要求，加强基层党组织建设，落实党风廉政建设主体责任和监督责任，认真学习贯彻廉洁自律准则和纪律处分条例，严格执纪问责，加强监督制约，以零容忍态度惩治腐败。做好2016年医院巡查工作，严肃查处违反医疗卫生行风建设“九不准”行为，塑造行业清风正气。细化落实中央八项规定精神的操作规定和标准，防止“四风”问题反弹。
　　（二十四）推进医学科技创新。贯彻落实国家创新驱动发展战略，着力构建人口健康科技创新体系，研究制订加快医药科技创新的指导意见。组织论证重大疾病国家实验室建设方案，继续组织实施国家科技重大专项，启动实施精准医学、出生缺陷干预等国家重点研发计划项目。建立国家生物安全工作协调机制。强化实验室生物安全监管。加强委级重点科研基地评估和动态管理。强化干细胞等医疗新技术临床研究管理，制订指导意见，推动卫生计生技术评估和适宜技术推广。
　　（二十五）强化财政资金保障。协调落实财政投入责任，继续加大对卫生计生事业的投入力度。加强中央财政转移支付地方项目资金管理，推进部门预算管理改革，开展经济责任审计，落实总会计师制度。做好价格和成本监测，加强国有资产和政府采购管理工作，强化项目与资金监管，组织开展绩效考评工作，提高资金使用效益。
　　（二十六）加强宣传引导和健康促进。加强正面宣传，发挥典型引领作用，大力弘扬卫生计生职业精神，做好节日纪念日宣传，推进健康文化和人口文化建设，启动婚育新风进万家活动第五阶段活动。大力倡导健康促进，加强健康科普规范化建设，深化健康中国行活动，扎实推进健康促进县（区）试点，办好第九届全球健康促进大会。积极控烟履约，推进无烟环境立法。加强舆情监测，完善全系统新闻发布制度，做好信息发布和政策解读，积极回应社会关切，提升突发事件舆论引导能力，营造有利于事业改革发展的社会舆论环境。
　　（二十七）深化国际合作交流。制订中国全球卫生战略，积极参与全球卫生治理，加强人口与发展领域合作。推进“一带一路”卫生交流合作。加强与非洲国家、周边国家、欧美等友好国家合作，着力经营好与非盟、东盟、中日韩、上海合作组织、金砖国家等卫生合作机制。帮助非洲加强公共卫生体系和能力建设。办好人口与发展南南合作伙伴组织部长级战略对话、第二届中国-中东欧卫生部长论坛、首届中国-东盟卫生合作论坛，落实中非卫生合作新举措，加强援外医疗队管理。
　　（二十八）进一步提升行政效能。加强中央决策部署和重点工作任务落实情况的督促检查，确保政令畅通。推进卫生计生系统社会信用体系建设，营造良好信用环境。落实重大决策社会稳定风险评估制度，加强和改进信访和调解工作，有效预防和化解矛盾纠纷。加强干部教育培训和能力建设。提高离退休人员服务管理水平。坚持管行业必须管安全，强化全行业安全生产监管职责和卫生计生机构主体责任，完善和落实安全生产规章制度，做好安全防范、隐患排查和督导通报。

© 北大法宝：（[www.pkulaw.com](https://www.pkulaw.com/)) 专业提供法律信息、法学知识和法律软件领域各类解决方案。北大法宝为您提供丰富的参考资料，正式引用法规条文时请与标准文本核对。
欢迎查看所有[产品和服务](https://www.pkulaw.com/helps/2.html)。法宝快讯：[如何快速找到您需要的检索结果？](https://www.pkulaw.com/helps/70.html)[法宝](https://www.pkulaw.com/helps/69.html)V6有何新特色？

扫描二维码阅读原文

**原文链接：**<https://www.pkulaw.com/chl/cb883063f5b3ebf6bdfb.html>
